# Comparative Research of GaN Growth Mechanisms on Patterned Sapphire Substrates with Sputtered AlON Nucleation Layers

**DOI:** 10.3390/ma13183933

**Published:** 2020-09-05

**Authors:** Yuan Gao, Shengrui Xu, Ruoshi Peng, Hongchang Tao, Jincheng Zhang, Yue Hao

**Affiliations:** State Key Discipline Laboratory of Wide Band Gap Semiconductor Technology, School of Microelectronics, Xidian University, Xi’an 710071, China; 15619217227@163.com (Y.G.); pengrs9@163.com (R.P.); hchtao@stu.xidian.edu.cn (H.T.); jchzhang@xidian.edu.cn (J.Z.); yhao@xidian.edu.cn (Y.H.)

**Keywords:** GaN growth, sputtered AlON nucleation layers, patterned sapphire substrates

## Abstract

The utilization of sputtered AlN nucleation layers (NLs) and patterned sapphire substrates (PSSs) could greatly improve GaN crystal quality. However, the growth mechanism of GaN on PSSs with sputtered AlN NLs has not been thoroughly understood. In this paper, we deposited AlON by sputtering AlN with O_2_, and we found that the variation of thickness of sputtered AlON NLs greatly influenced GaN growth on PSSs. (1) For 10 nm thin AlON sputtering, no AlON was detected on the cone sidewalls. Still, GaN nucleated preferably in non-(0001) orientation on these sidewalls. (2) If the thickness of the sputtered AlON NL was 25 nm, AlON formed on the cone sidewalls and flat regions, and some small GaN crystals formed near the bottom of the cones. (3) If the sputtered AlON was 40 nm, the migration ability of Ga atoms would be enhanced, and GaN nucleated at the top of the cones, which have more chances to grow and generate more dislocations. Finally, the GaN growth mechanisms on PSSs with sputtered AlON NLs of different thicknesses were proposed.

## 1. Introduction

GaN-based light emitting diodes (LEDs) and laser diodes (LDs) have made huge progress in recent years [1,2,3]. To further enhance the performance of optoelectronic devices, patterned sapphire substrates (PSSs) are widely used to strengthen the light extraction efficiency (LEE) and external quantum efficiency [4,5,6]. The utilization of PSSs could greatly annihilate dislocations [4,7,8,9]. Interestingly, with the appropriate nucleation layer (NL), most GaN grows in the flat regions between cones, though the flat region is a rather small proportion of the whole substrate, no matter what the geometry of the pattern is [10,11,12]. However, some GaN would still form small GaN crystals on the cone sidewalls, which may have negative influences on GaN growth [13,14]. In our previous work, we confirmed that small GaN crystals would generate dislocations [15]. When using metal organic chemical vapor deposition (MOCVD) to grow AlN or GaN NLs, the influences of small GaN crystals become more noteworthy [13,14,16]. Compared to MOCVD-grown AlN or GaN NLs, Li-Chuan Chang et al. found that the utilization of ex situ sputtered AlN NLs could suppress GaN nucleation on cones and improve the quality of GaN films on PSSs [17]. Subsequently, many works proved that the utilization of sputtered AlN/PSS templates could improve the performance of GaN-based LEDs [18,19]. However, small GaN crystals on the cones’ surface of sputtered AlN/PSS templates may still deteriorate the crystal quality [15,20,21]. The study of small GaN crystals on sputtered AlN/PSS templates is beneficial to further improve the crystal quality.

In this work, we deposited AlON on PSSs by sputtering AlN with small flux feeding of O_2_, which could improve the quality of the GaN film. We found that the thickness of the sputtered AlON NL had great influences on GaN growth. When the sputtered AlON NL was 10 nm, large crystals nucleated on the cone sidewalls, and GaN grew in non-(0001) orientation. When the sputtered AlON NL was 25 nm, small GaN crystals formed near the bottom of the cones, which merged at early stages of growth. As GaN grew on 40 nm sputtered AlON/PSS templates, GaN crystals formed at the top of the cones, and the crystals had more opportunities to grow. The disparity of the small GaN crystals on cones resulted in the different crystal quality of GaN films. After detailed characterization and analysis, we obtained the optimum thickness of sputtered AlON and illustrated the GaN growth mechanism with sputtered AlON/PSS templates.

## 2. Materials and Methods

The 2 in c-plane cone-shaped PSSs (bottom diameter, interval spacing, and height of the cones are 2.7, 0.2, and 1.5 μm) were prepared by inductively coupled plasma etching and photolithography. After the preparation of PSSs, AlON layers were sputtered on the substrates at 650 °C by feeding 2 sccm O_2_, 30 sccm Ar_2_, and 180 sccm N_2_. The thickness of AlON was based on the sputtering technique on conventional sapphire substrates (CSSs) by the mature commercial sputtering system. Before GaN growth, the sputtered AlON/PSS templates were pre-treated under an atmosphere of H_2_ for 5 min, and the temperature was 1100 °C. After that, the templates were cooled down to 520 °C, and the nitridation of the templates was under an atmosphere of NH_3_ for 3 min. Then, 5 μm GaN was grown on 10, 25, and 40 nm sputtered AlON/PSS templates. Trimethylgallium and ammonia were the precursors for Ga and N. High-purity N_2_ was employed as the carrier gas, and the pressure of the reaction chamber was held at 400 mBar. In the first step (900 s), the growth temperature was 970 °C, and the V/III mole ratio was 1500. In the second step (500 s), the temperature and V/III mole ratio were gradually varied to 1060 °C and 900. During the third step (4000 s), the temperature and V/III mole ratio were held at 1060 °C and 900. To further figure out the differences of the growth process between three samples, we did another group of experiments on conventional sapphire substrates (CSSs) with 10, 25, and 40 nm sputtered AlON layers. The growth condition of GaN films on CSSs remained the same as that on PSSs.

## 3. Results and Discussion

### 3.1. Charatcerization of Sputtered AlON/PSS Templates

**Figure 1:**
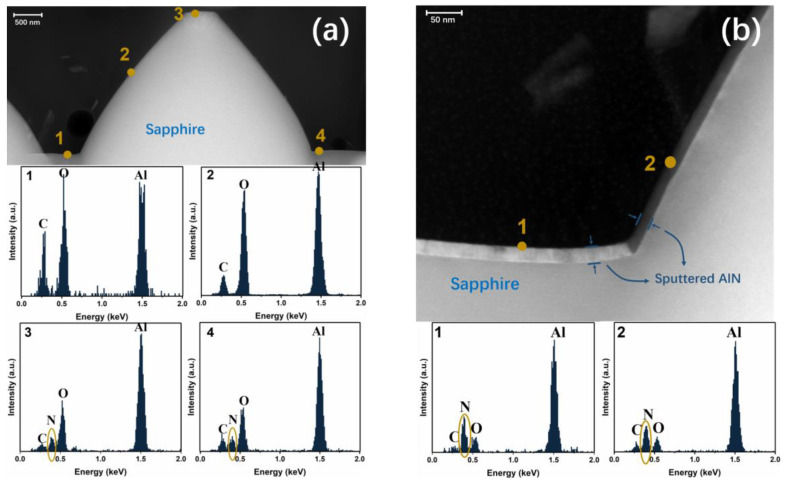
(**a**) The TEM image of the 10 nm sputtered AlON/PSS template and EDX results of four spots. Nitrogen is only found in spots 1 and 4. (**b**) The TEM image of the 25 nm sputtered AlON/PSS template and EDX results of two spots.

Firstly, we made a detailed characterization of 10 nm and 25 nm sputtered AlON/PSS templates. The cross-sectional transmission electron microscope (TEM) images and energy-dispersive X-ray spectroscopy (EDX) were acquired by ecnai G2 F20 S-Twin operating at 200 kV. The cross-sectional TEM and EDX results are provided in Figure 1. We marked some spots in Figure 1 that matched the EDX results. In Figure 1b, the sputtered AlON layers in the flat regions and cones were 23.3 nm and 17.1 nm, respectively. The thickness of AlON was based on the standard of the sputtering technique on CSSs, so the thickness of the sputtered AlON layer on the PSS had some deviation, which was also reported by other groups [4,16]. The EDX results of four spots in the 10 nm sputtered AlON layer are given in Figure 1a. Nitrogen was only found in the flat regions, which means there were no effective NLs on the cone sidewalls. As for the 25 nm sputtered AlON/PSS template, nitrogen could be found in both flat regions and the cones’ surface.

### 3.2. Morphology Comparation at Early Stages

**Figure 2:**
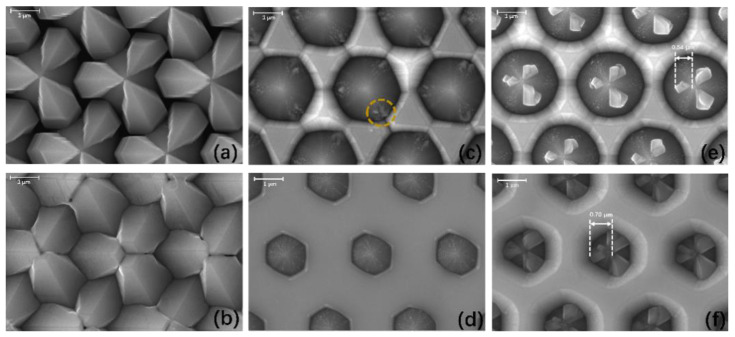
Top-view SEM images of GaN films on (**a**) 10 nm, (**c**) 25 nm, and (**e**) 40 nm sputtered AlON/PSS templates at stage 1; GaN films on (**b**) 10 nm, (**d**) 25 nm, and (**f**) 40 nm sputtered AlON/PSS templates at stage 2.

Subsequently, we obtained the morphology information of GaN films on 10, 25, and 40 nm sputtered AlON/PSS templates at two early stages, named stage 1 (200 s) and stage 2 (800 s). The top-view images were captured by FEI MLA 650F scanning electron microscope (SEM). From Figure 2a, large GaN crystals formed on the cones, and we could not find any GaN in the flat regions, which means GaN mainly nucleated and grew on the cones. At stage 2, the collision of large GaN crystals generated many interfaces, and the GaN film could not coalesce, observed in Figure 2b. If the thin, low-temperature MOCVD GaN NL was deposited on the PSS, or there was no NL on the cones of the PSS, GaN mostly nucleated on the cones [6,19,22].

Compared with the GaN film on the 10 nm sputtered AlON/PSS template, GaN grew from the flat regions on 25 nm and 40 nm sputtered AlON/PSS templates. At stage 1, there was only small GaN on the cones, observed from Figure 2c,e. Interestingly, the small GaN crystals on the 25 nm and 40 nm sputtered AlON/PSS templates were at different positions on the cones’ surface. Small GaN crystals in Figure 2c were near the bottom of the cones, and they did not have a clear shape. The small GaN crystals on the 40 nm sputtered AlON/PSS template were at the top of the cones and had clearer shapes. At stage 2, small GaN crystals on the 25 nm sputtered AlON/PSS template were totally merged, but we could still see the GaN crystals on the 40 nm sputtered AlON/PSS template. The size of GaN crystals on the 40 nm sputtered AlON/PSS template increased from 0.54 μm to 0.70 μm by our measurement. It could be inferred that GaN crystals on the 40 nm sputtered AlON/PSS template had more chances to grow, and generated more dislocations [15,20,21].

**Figure 3:**
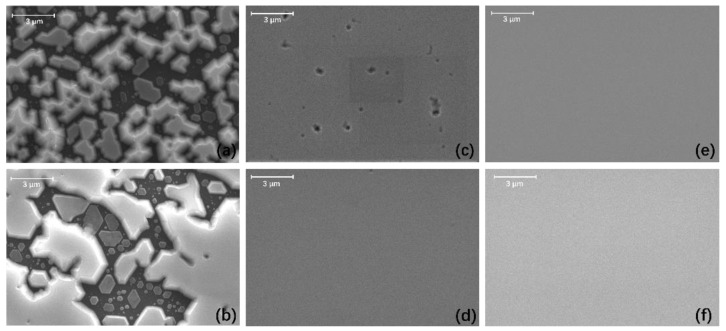
Top-view SEM images of GaN films on (**a**) 10 nm, (**c**) 25 nm, and (**e**) 40 nm sputtered AlON/CSS templates at stage 1; GaN films on (**b**) 10 nm, (**d**) 25 nm, and (**f**) 40 nm sputtered AlON/CSS templates at stage 2.

When the thickness of sputtered AlON turned from 25 nm to 40 nm, small GaN crystals nucleated from the bottom to the top of the cones. The thickness of sputtered AlON may have had influences on the migration ability of Ga atoms. Stronger migration ability of Ga atoms would have enhanced lateral growth, and the islands would have coalesced earlier, which has been proven in other nitride materials like AlN [23,24]. Therefore, we grew GaN films on CSSs with 10, 25, and 40 nm sputtered AlON. We obtained top-view SEM images of GaN films on 10, 25, and 40 nm sputtered AlON/CSS templates at early stages in Figure 3.

We observed that the GaN film on the 40 nm sputtered AlON/CSS template had totally coalesced at stage 1, from Figure 3e. Compared with the GaN film on the 40 nm sputtered AlON/CSS template, there were some holes on the surface of the GaN film on the 25 nm sputtered AlON/CSS template. Therefore, GaN grown on the 40 nm sputtered AlON/CSS template coalesced earlier than that on the 25 nm sputtered AlON/CSS templates, which meant the migration ability of Ga atoms on the 40 nm sputtered AlON was stronger. Therefore, the stronger migration ability promoted Ga atoms to move to the top of the cones and form GaN. However, for sample B, the small GaN crystals could only form near the bottom of the cones because of the weaker migration ability of Ga atoms, shown in Figure 2c,e.

In the GaN film on the 10 nm sputtered AlON/PSS template, the GaN film could not coalesce even at stage 2. Thus, we concluded that the migration ability of Ga atoms on the 10 nm sputtered AlON layer was relatively weak, compared with Ga atoms on the 25 or 40 nm sputtered AlON layers. The weak migration ability of GaN atoms on 10 nm sputtered AlON made lateral growth of GaN islands relatively slower than GaN islands on the 25 or 40 nm sputtered AlON/PSS template.

### 3.3. Charaterizaiton of the GaN Films

**Figure 4:**
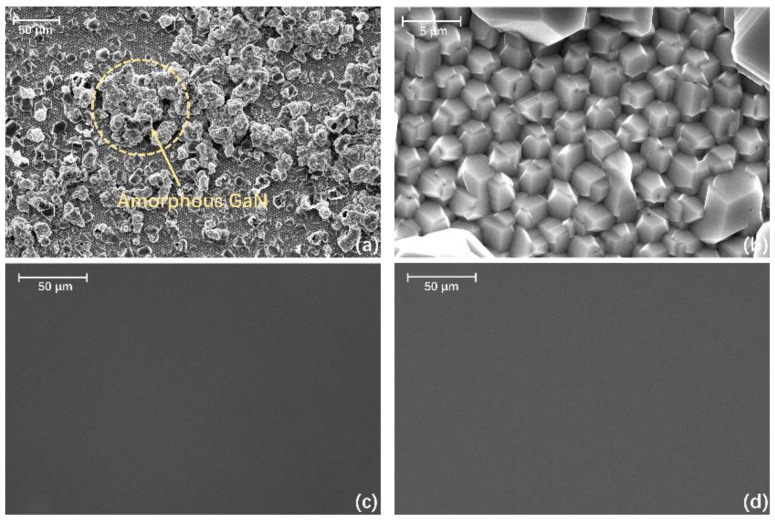
(**a**)The top-view SEM images of the GaN film on the 10 nm sputtered AlON/PSS template and (**b**) a partly magnified top-view SEM image. Smooth morphology of GaN films on (**c**) the 25 nm and (**d**) 40 nm sputtered AlON/PSS. GaN film on 10 nm sputtered AlN/PSS template does not coalesce well and some amorphous GaN appears. The morphology of the GaN on 25 nm and 40 nm sputtered AlN/PSS templates.

We have confirmed that the thickness variation of sputtered AlON NLs had great influences on the GaN growth on PSSs at early stages. To further discuss the GaN films, we did detailed characterization of 5 μm GaN films on 10, 25, and 40 nm sputtered AlON/PSS templates. Figure 4 gives the top-view SEM images of GaN films on 10, 25, and 40 nm sputtered AlON/PSS templates. We observed that the surface of GaN films on 25 nm and 40 nm sputtered AlON/PSS templates was very smooth from Figure 4c,d. However, for the GaN film on the 10 nm sputtered AlON/PSS template, GaN could not coalesce and some amorphous GaN formed, shown in Figure 4b.

**Figure 5:**
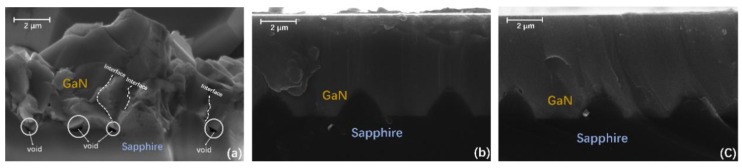
The cross-sectional SEM images of GaN films on (**a**) 10 nm, (**b**) 25 nm, and (**c**) 40 nm sputtered AlON/PSS templates.

The cross-sectional SEM images of GaN films on 10, 25, and 40 nm sputtered AlON/PSS templates were taken from the [11,12,13,14,15,16,17,18,19,20] direction, presented in Figure 5. From the cross-sectional SEM image of the GaN film on the 10 nm sputtered AlON/PSS template, we could clearly observe that GaN could not coalesce well. In Figure 5a, we saw that there were voids between the cones, and the interfaces resulted from the collision of GaN crystals. As for GaN films on 25 nm and 40 nm sputtered AlON/PSS templates, there were no hollows or cracks.

**Figure 6:**
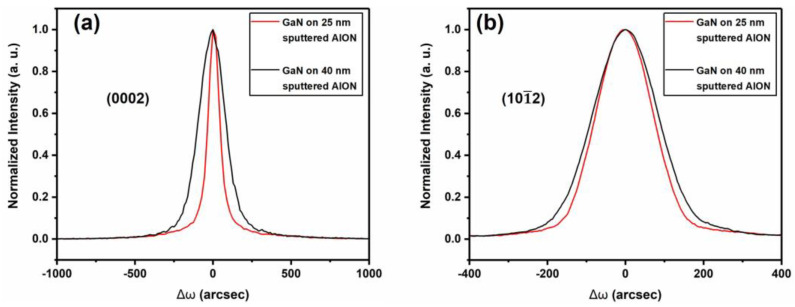
ω-scan rocking curve at the symmetric (**a**) (0002) plane and (**b**) (10$\bar{1}$2) plane of GaN films on 25 nm and 40 nm sputtered AlON/PSS templates.

Although the morphology of GaN films on the 25 nm and 40 nm sputtered AlON/PSS was very smooth, we considered that the crystal quality of the GaN film on the 40 nm sputtered AlON/PSS would be inferior to that on the 25 nm sputtered AlON/PSS template, for the larger GaN crystals on the cone sidewalls generated more dislocations. To verify our analysis, we characterized the crystal quality of GaN films on 10, 25, and 40 nm sputtered AlON/PSS templates by Bruker D8 high-resolution X-ray diffraction (HRXRD). The full width at half maximum (FWHM) of the ω-scan rocking curve at the symmetric (0002) and (10$\bar{1}$2) plane can reflect the dislocation density [25,26]. Because the quality of sample A was severely deteriorated, the rocking curve of sample A could not be obtained. Figure 6 and Table 1 give the ω-scan rocking curve and calculated dislocations of GaN films on 25 nm and 40 nm sputtered AlON/PSS templates.

FWHM results of the ω-scan rocking curve at the symmetric (0002)/(10$\bar{1}$2) planes of GaN films on 25 nm and 40 nm sputtered AlON/PSS templates were 87/149 arcsec and 196/169 arcsec, respectively. The calculated dislocation densities of GaN films on 25 nm and 40 nm sputtered AlON/PSS templates are presented in Table 1. The density of total dislocations in the GaN film on the 40 nm sputtered AlON/PSS template was about 70% higher than that in the GaN film on the 25 nm sputtered AlON/PSS template. Thus, we concluded that when GaN grew on the 40 nm sputtered AlON/PSS templates, the larger crystals at the top of the cones generated more dislocations.

**Figure 7:**
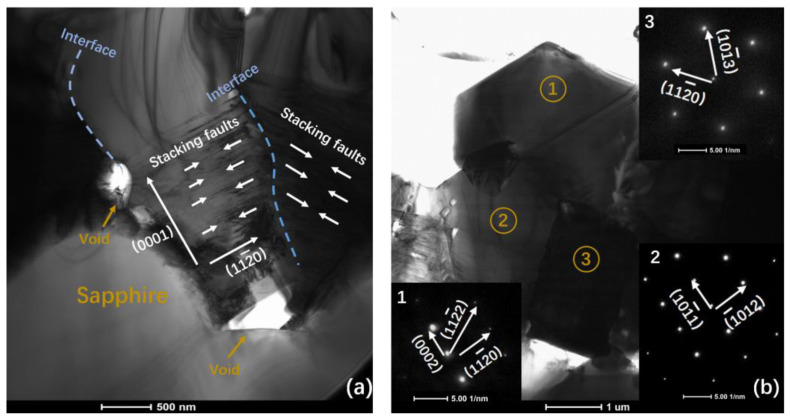
(**a**) The STEM image of the GaN film on the 10 nm sputtered AlON/PSS. (**b**) SAED patterns of the GaN film on the 10 nm sputtered AlON/PSS template.

The growth process of the GaN film on 10 nm sputtered AlON/PSS was complicated, so we used cross-sectional TEM to further determine the characteristics. Figure 7 provides detailed information of the GaN film on 10 nm sputtered AlON/PSS, and selected-area electron diffraction (SAED) patterns are also presented. From Figure 7a, we could clearly see that GaN nucleated on the cones. GaN grew larger from the cones’ surface and collided with each other. The slope of the sapphire cones allowed for tilted nucleation of a-plane GaN similar to a-plane growth on r-plane sapphire [27]. The stacking faults indicated that GaN on the cone sidewalls presented a-plane orientation [28,29,30,31]. We observed that there were some semi-polar and non-polar planes in sample A, by our calculation from SAED patterns in Figure 7b.

**Figure 8:**
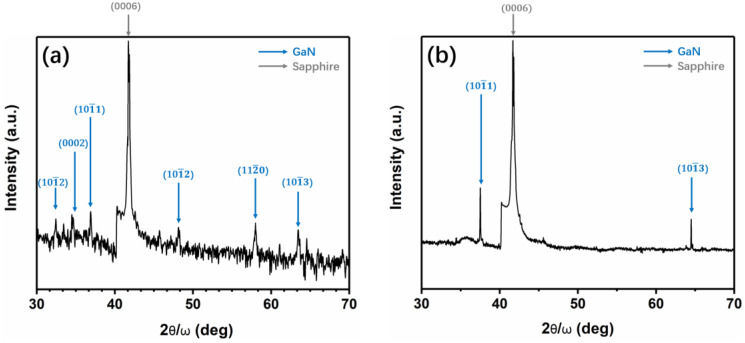
A symmetric (0004) 2θ/ω scan for (**a**) the GaN film on 10 nm sputtered AlON/PSS and (**b**) the GaN film on 10 nm sputtered AlON/PSS at stage 2.

We performed a symmetric (0004) 2θ/ω powder XRD characterization for sample A to verify of our SAED calculation by Bruker D8 advance ECO, shown in Figure 8. We inferred that GaN grew in non-(0001) orientation. The powder XRD results of the GaN film on the 10 nm sputtered AlON/PSS matched the calculation results from SAED patterns.

### 3.4. GaN Growth Mechanisms on Sputtered AlON/PSS Templates

Because there were no effective NLs on the cones of the 10 nm sputtered AlON/PSS, GaN mostly nucleated on the cones and formed large non-(0001) GaN crystals under the experimental conditions. As the growth proceeded, GaN crystals grew larger and collided with each other, which generated many interfaces. GaN could not coalesce and some amorphous GaN appeared.

If the sputtered AlON was 25 nm, GaN nucleated in the flat regions, and the coalescent process was successful. However, some Ga atoms inevitably transferred to the cones and formed small GaN crystals. Compared with 40 nm sputtered AlON/PSS templates, the small GaN crystals were near the bottom of the cones. Small GaN crystals merged in a short time of growth, which is shown in Figure 2d. Moreover, the small GaN crystals on cones had fewer chances to grow larger and generate dislocations.

For GaN film grown on the 40 nm sputtered AlON/PSS template, the strong migration ability on 40 nm sputtered AlON/PSS templates meant GaN crystals could nucleate at the top the cones. Therefore, it needed more time for the small GaN crystals to merge and have more chances to grow and generate dislocations.

## 4. Conclusions

In this paper, we systematically discussed the differences of GaN growth mechanisms on PSSs with sputtered AlON NLs of different thickness. When the sputtered AlON was 10 nm, there were no effective sputtered AlON layers on the cones’ surface. Large non-(0001) GaN crystals nucleated on the cones, and they could not coalesce during growth. When the thickness of the sputtered AlON NL was 25 nm or 40 nm, GaN mainly grew from the flat regions, and only small GaN crystals formed on the cones’ surface. Our experiment on sputtered AlON/CSS templates showed that the migration ability of Ga atoms on the 40 nm sputtered AlON NL was stronger than that on the 25 nm sputtered AlON NL. Therefore, compared with GaN film on the 25 nm sputtered AlON/PSS template, the small GaN crystals on 40 nm sputtered AlON/PSS templates formed at the top of the cones, which had more time to grow and generate more dislocations. The larger GaN crystals on 40 nm sputtered AlON/PSS templates deteriorated the crystal quality of the GaN film. Finally, we gave the mechanisms of GaN growth on sputtered AlON/PSS templates, as the thickness of sputtered AlON varied from 10 nm to 40 nm.

## Figures and Tables

**Table 1:** FWHM results of the ω-scan rocking curve at the symmetric (0002) and (10$\bar{1}$2) planes, and calculated density of dislocations.

Sample	FWHMs (0002)/(10$\bar{1}$2)	Density of Screw Dislocations	Density of Edge and Mixed Dislocations	Density of Total Dislocations
B	87/149 arcsec	1.51 × 10^7^ cm^−2^	1.13 × 10^8^ cm^−2^	1.33 × 10^8^ cm^−2^
C	196/169 arcsec	7.70 × 10^7^ cm^−2^	1.49 × 10^8^ cm^−2^	2.25 × 10^8^ cm^−2^
